# Supplementary material for: Quantitative real-time in-cell imaging reveals heterogeneous clusters of proteins prior to condensation
Source: Nat Commun. 2023 Aug 15;14:4831. doi: 10.1038/s41467-023-40540-2 (PMC10427612; doi:10.1038/s41467-023-40540-2)
Supplement: Supplementary file 1 — Supplementary Information [file 41467_2023_40540_MOESM1_ESM.pdf]

Supplementary Information:
Quantitative real-time in-cell imaging reveals heterogeneous clusters of proteins prior to condensation

Chenyang Lan,[1,2,3] Juhyeong Kim,[1] Svenja Ulferts,[4] Fernando Aprile-Garcia,[5] Sophie Weyrauch,[1,6,7] Abhinaya Anandamurugan,[1] Robert Grosse,[4] Ritwick Sawarkar,[8] Aleks Reinhardt,[9,*] and Thorsten Hugel[1,2,*]
[1]*Institute of Physical Chemistry, University of Freiburg, Freiburg, Germany*
[2]*BIOSS and CIBSS Signalling Research Centres, University of Freiburg, Freiburg, Germany*
[3]*PicoQuant GmbH, Rudower Chaussee 29, 12489 Berlin, Germany*
[4]*Institute of Experimental and Clinical Pharmacology and Toxicology, University of Freiburg, Germany*
[5]*Max Planck Institute of Immunobiology and Epigenetics, Germany*
[6]*Spemann Graduate School of Biology and Medicine (SGBM), University of Freiburg, Freiburg, Germany*
[7]*Faculty of Chemistry and Pharmacology, University of Freiburg, Freiburg, Germany*
[8]*Medical Research Council (MRC), University of Cambridge, Cambridge, CB2 1QR, United Kingdom*
[9]*Yusuf Hamied Department of Chemistry, University of Cambridge, Cambridge, CB2 1EW, United Kingdom*

(Dated: 12th July 2023)

Note 1: Measurement of the ratio of NELFA-GFP to endogenous NELFA

HeLa cells were cultured in six-well plates as mentioned in the Methods section, and NELFA-GFP expression was induced by tetracycline ($1\,\mu g\,mL^{-1}$) for four and six hours. Cells were harvested and washed in PBS. Cell pellets were frozen in liquid nitrogen and stored at $-80\,°C$ until Western-blot analysis. Cell pellets were thawed gradually on ice and lysed in lysis buffer (50 mM Tris-HCl pH 6.8, 2 % SDS and 10 % glycerol). Cellular lysates were analysed by SDS-PAGE followed by immunoblotting using antibody for NELFA (1 : 500; Santa Cruz sc-23599). Signals were quantified by densitometry using Image Lab (Bio-Rad Laboratories).

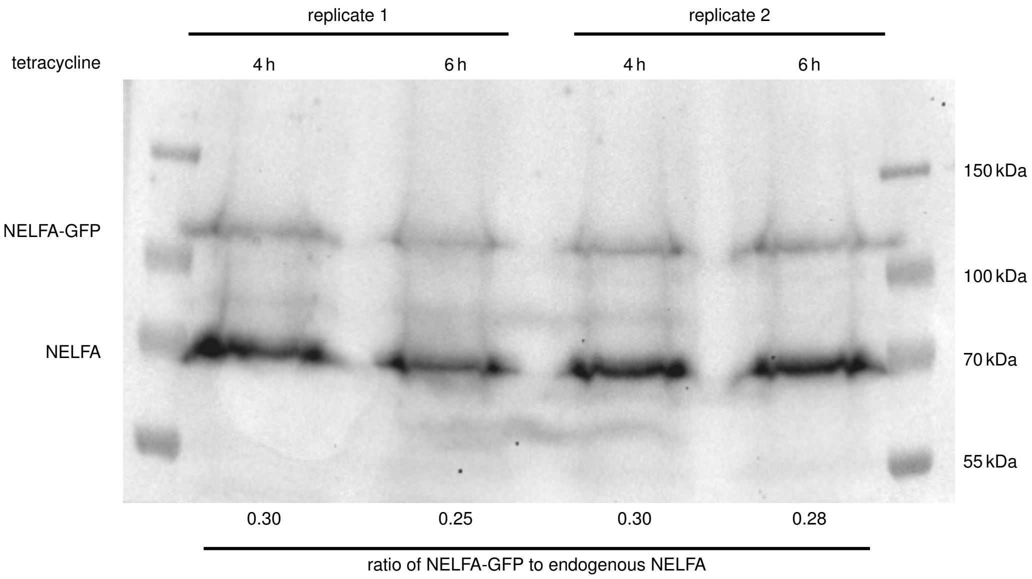

Figure S1 Western-blot analysis to measure the ratio of NELFA-GFP to endogenous NELFA. NELFA-GFP expression was induced with tetracycline for 4 h to 6 h. Two replicates were run in the same gel. Endogenous NELFA serves as a protein loading control. Molecular mass markers are also shown, with masses indicated on the right. Different camera set-ups are needed for protein bands (luminescence) and mass markers (visible); the same gel was photographed in sequence and the two photographs merged. An unmerged version of the protein bands is included in the supporting data.[1]

*Correspondence to: ar732@cam.ac.uk and th@pc.uni-freiburg.de

Note 2: Measurement of the viability of HeLa-NELFA by trypan blue assay

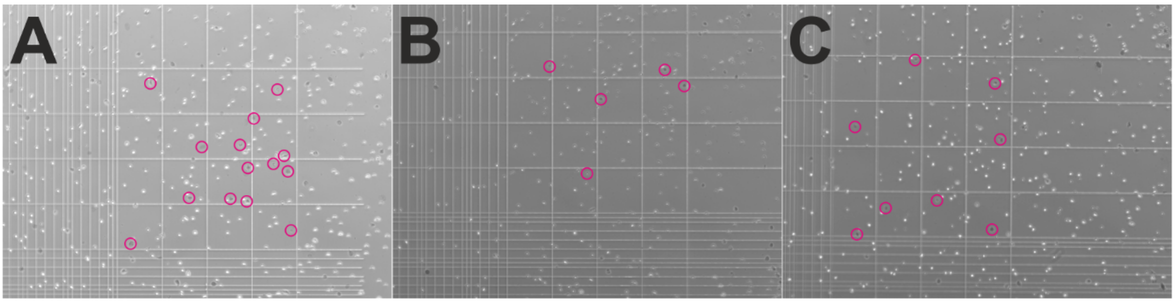

Figure S2 Representative bright-field images of fresh and As_2O_3-treated HeLa-NELFA cells stained by trypan blue. Living and adherent HeLa-NELFA cells were washed by DPBS, detached by trypsin, centrifuged (100*g*, 5 min, 4 ℃), and resuspended in 1 mL DPBS. 100 µL suspended cells were treated by 100 µL trypan blue for 3 min. Bright (living) and dark (dead, marked by circles in magenta) cells were imaged and counted in a haemocytometer. **a** The viability ratio in fresh cells was 86 %. **B** The viability ratio in cells following a 1 h As_2O_3 treatment of cells growing in Invitrogen living-cell imaging solution was 95 %. **c** After As_2O_3 treatment, cells were washed by DPBS three times and grown in cell culture media for 24 h. The viability of cells after this treatment was 94 %.

Note 3: Setup and living-cell imaging of synthetic NELFA-GFP at high expression levels and synthetic 2NT-DDX4-GFP

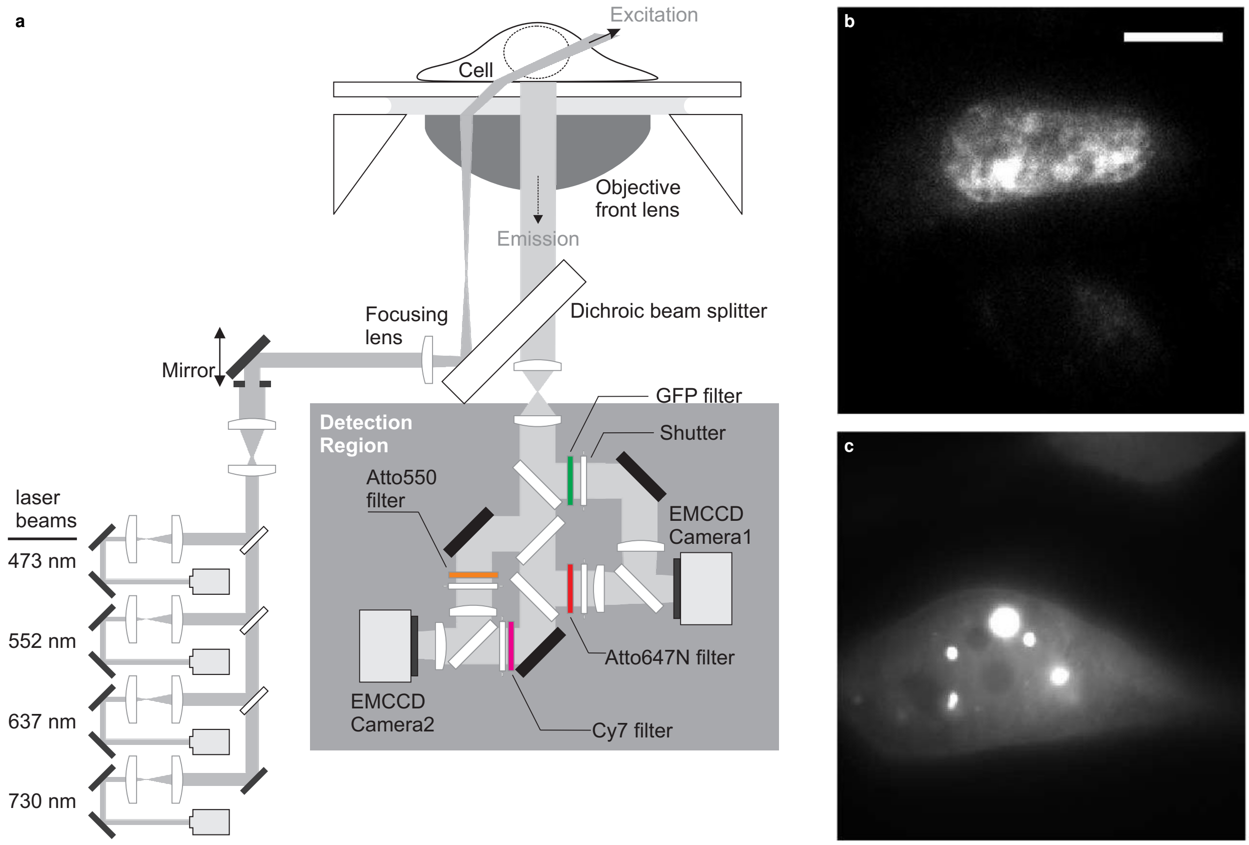

Figure S3 **a** Schematic illustration of the custom-built TIRF/HILO microscope for single-molecule imaging or biological phase separation in living cells. **b** A living HeLa cell expressing the NELFA-GFP (24 h of expression time) followed by 1 h of arsenic exposure (one snapshot from 3 movies). **c** A living HeLa cell expressing synthetic 2NT-DDX4-GFP (6 h after transfection)[2] undergoing phase separation using the TIRF/HILO microscope. With this experiment we show that our setup is capable of resolving several spherical clusters with our HILO microscope, if such clusters are present[2] (snapshot from 1 movie). Scale bar 10 µm.

We usually only observe the formation of a single (final) condensate. At first, this may appear to contradict previous observations of several condensates for NELFA[3] as well as other publications on different proteins. In order to make sure that we do not miss other condensates in the cell, in other imaging planes, we recorded 3D Airyscan movies and confirmed that there was indeed only one large condensate under the low NELFA-GFP expression conditions used here. The formation of a single condensate is consistent with a nucleation-and-growth model requiring a critical nucleus size of around 2000 NELFA molecules. As the first post-critical nucleus grows quickly, it depletes the reservoir of NELFA molecules in the system, leaving insufficient molecules to

form a second post-critical nucleus. By contrast, at the expression conditions used in our previous publication,[3] there are several times more proteins in the cell, which suffices to form several condensates.

Note 4: Machine-learning-based tracking of irregular shapes of NELFA-GFP regions

In our living-cell imaging results, images in movies had a low signal-to-noise ratio as a trade-off to avoid significant photobleaching of GFPs. To segment regions of NELFA-GFP better, we resorted to a combination of machine-learning-based segmentation algorithms, trainable Weka segmentation (Weka), a Fiji plugin (https://imagej.net/plugins/tws/how-to-install-new-classifiers).[4] First, we set three training features (Gaussian blue, Hessian and Membrane projections). Next, we set three classifiers to determine regions of NELFA-GFP, regions of cellular background, and regions of non-cellular background. By manually delineating borders of each type of region in several frames, we trained the Weka until the machine-learning-based recognition of regions was at the level of eye detection. The output of Weka was a movie comprising binary images for each type of region.[1]

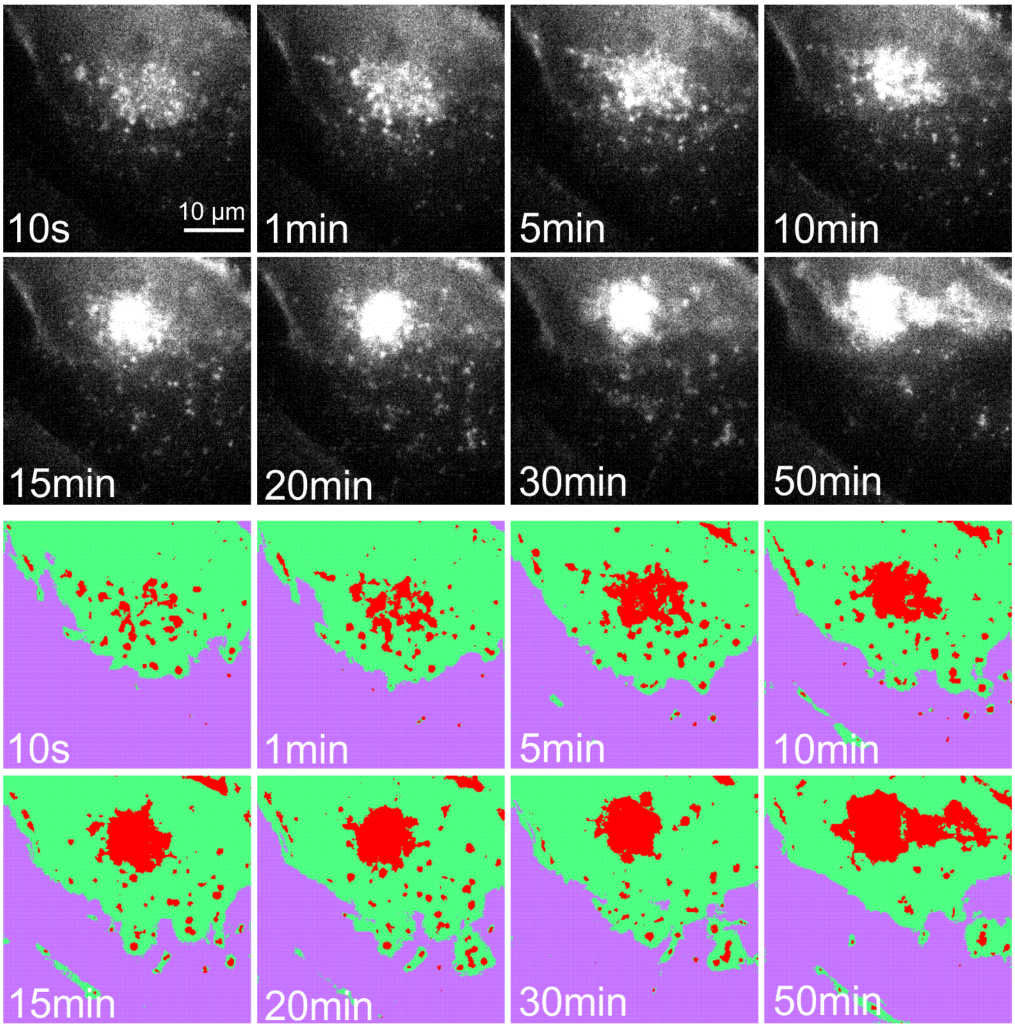

Figure S4 Machine-learning-based segmentation of images with trainable Weka segmentation. In eight images following the training by Weka, red regions are NELFA-GFP, green regions are regions of cellular background, and purple regions are regions of non-cellular background, respectively.

Once NELFA-GFP regions from Weka were obtained without backgrounds, we further used MOSAICsuite, another plugin in Fiji, to track NELFA-GFP regions automatically, accounting for the constantly changing region shapes. To enable reliable tracking, we first processed the binary images of NELFA-GFP regions in the sub-plugin Squassh in MOSAICsuite to perform a post-segmentation for the subsequent MOSAIC tracking.[5,6] We used the following setting in Squassh:

1. in Background Subtraction, disable 'Remove Background';
2. Regularization (>0) ch1 & ch2 = 0.2 and Minimum Object Intensity Channel 1 & 2 = 0.3 for most movies;
3. Select the 'Exclude Z edge' option;
4. Select 'Automatic' in Local Intensity Estimation and 'Poisson' in Noise Model;

5. Values are all set to be 1 in the PSF Model;
6. 'Remove Region with Intensities' should be less than zero and 'Remove Region with Size' should be less than 2.

The Particle Tracker 2D/3D further processed the output of Squassh in the MOSAICSuite with Link Range set to 2 and Displacement set to 20, respectively.[6]

Note 5: Representative data sets of cluster sizes over time in living stressed and unstressed cells

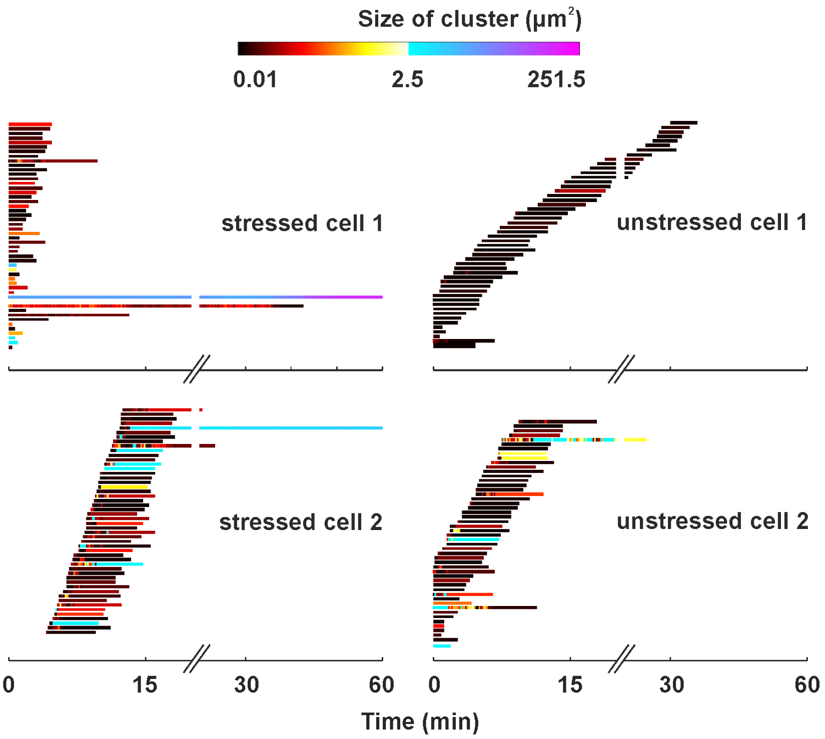

Figure S5 Kymographs of two stressed and two unstressed living cells showing changes in sizes of clusters over time. All cells show dynamic sizes, but only the stressed cells show one cluster each growing to a size of more than 10 μm^2.

Note 6: Automated photobleaching-step counting in fixed HeLa cells

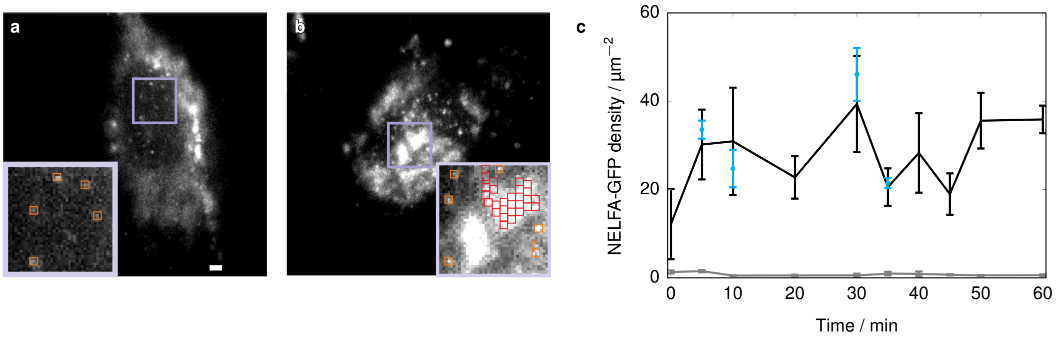

Figure S6 Converting areas of GFP regions into numbers of NELFA-GFP molecules. **a** Isolated GFP spots found in an unstressed and fixed HeLa cell. In the zoomed-in panel, five regions are outlined in orange. **b** Isolated and contiguous GFP regions found in a stressed and fixed HeLa cell. The condensate (i.e. the largest contiguous region) corresponds to regions outlined in red. Scale bar: 2 μm. Photobleaching steps were measured from each grid. **c** Density of NELFA-GFP molecules in dense (black) and dilute (grey) phases measured in fixed cells. Data from 4330 photobleaching traces and a total of 35 cells. Error bars show the standard error of the mean across 35 fixed cells. The average number density of NELFA-GFP in the dense phase is (29 ± 2) μm^{-2}. As a second method of determining the NELFA-GFP density, we have performed a brightness analysis of cluster sizes. Specifically, we determined the total brightness of every 3×3 grid in the largest cluster and divided this brightness by the mean intensity of single photobleaching steps, which can be very readily resolved [see Fig. 2a (top)]. These results are displayed in cyan and are consistent with the photobleaching step analysis. Each error bar in cyan gives the standard deviation in this value computed across 10 grids.

To count the number of NELF-GFP molecules, we measured photobleaching steps from nuclear GFP regions by imaging HeLa cells over time (200 s or 400 s) and photobleached GFP (with a 473 nm laser power of 2 mW measured at the laser set).

In a movie, GFP regions first appeared as either isolated spots or contiguous regions [Fig. S6a–b]. To extract stepwise photobleaching curves, we first set a threshold such that most isolated spots appeared with a size of 3 × 3 pixels in the first frame of the movie. This is because the theoretical diameter of an Airy disk of one GFP spot is $1.22 \times 509\,\text{nm}/1.49 = 417\,\text{nm}$, with the fluorescence being at ~509 nm and the numerical aperture of the objective being 1.49. This is about three pixels in one image (one pixel = 160 nm × 160 nm).

For each photobleaching trace, photobleaching steps were determined by AutoStepfinder [Fig. 2a].[7] Steps from AutoStepfinder should be consistent with those determined by hand by observing multiple simulated traces with various steps. The value of photobleaching steps is the maximal number of NELF-GFP molecules in each grid. The quantification of the number of NELF-GFP from the photobleaching step analysis was double-checked by an intensity-based analysis (see Fig. S6c and its caption).

We define the 'dilute phase' of NELF as the region where only isolated spots with a size of 3 × 3 pixels were present, and the 'dense phase' as the largest contiguous GFP region with a size larger than 3 × 3 pixels). In other words, this is the largest phase-separated domain within a HeLa nucleus.

In the dense phases, the number density of NELF-GFP molecules can be calculated as the total number of molecules (determined by the total number of photobleaching steps) divided by the total area of the grids, $A_{\text{cluster}} = N_{\text{grids}} \times 0.23\,\mu\text{m}^2$. For dilute phases, the density of NELF-GFP molecules is the total number of steps for all dilute-phase regions divided by the total area of the nuclear region ($4\pi r^2_{\text{nucleus}} = 4\pi(3\,\mu\text{m})^2 = 113.04\,\mu\text{m}^2$), with the area of the dense phase subtracted.[2]

Note 7: Analysis of the onset of NELFA-GFP condensation in living-cell imaging movies

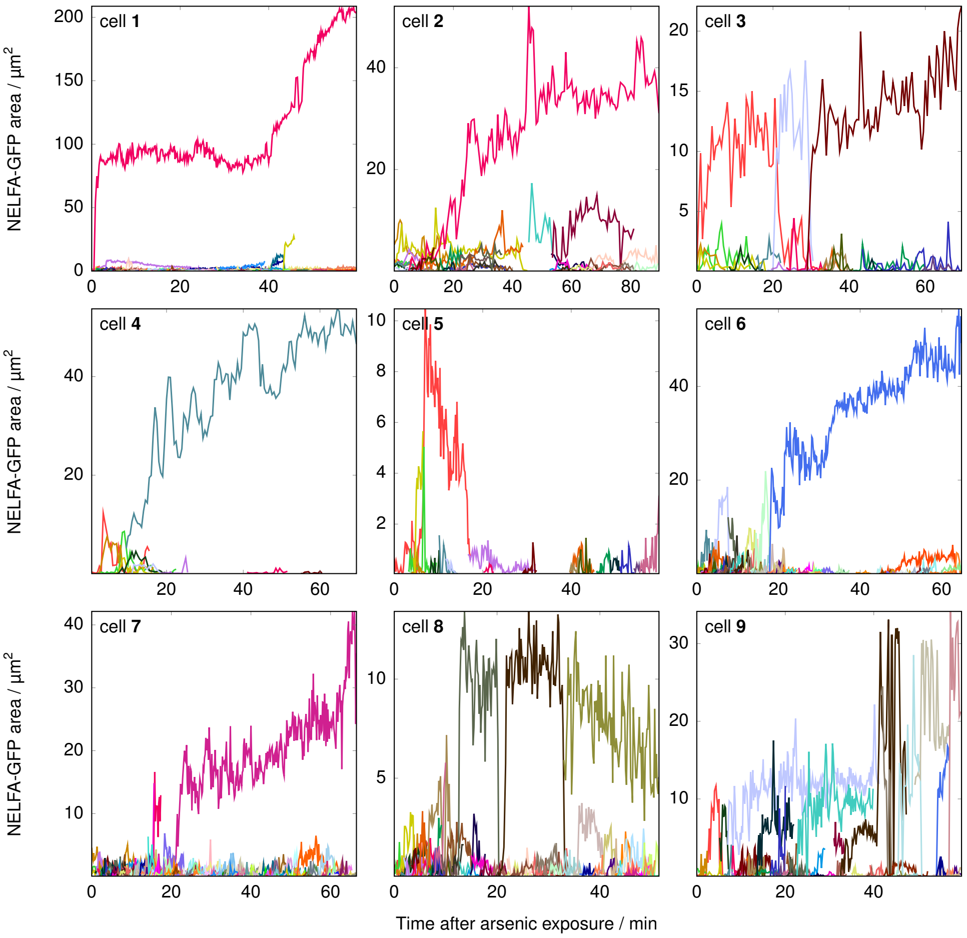

Figure S7 Changes in cluster sizes after arsenic exposure from the regions of interest for all cells shown in Supplementary Movie 4.[1] Each colour represents one cluster. In total, 3631 clusters were tracked. For Fig. 2d and Fig. 2e, the choices of threshold times to delineate pre- and post-nucleation behaviour for each cell are 0.4 min (cell1), 21 min (cell2), 30 min (cell3), 13 min (cell4), 22 min (cell6), 21 min (cell7) and 7 min (cell9), respectively. For cell9, the growth into a condensate is less clear, but we nevertheless used the time for the cluster in a blue hue (which grows at around the 7 min mark) because this cluster remains stable in size until about 40 minutes, which is a much longer lifetime than that of any other small cluster.

Note 8: Effective diffusion coefficients and gradients in cluster size

We show in Fig. S8a the effective diffusion coefficient $D(j)$ (computed by a fit to the Einstein relation,[8] $\langle r^2 \rangle = 4Dt$) and size gradient $\nabla_{\text{size}}(j)$ for every cluster j; the two results are only very weakly correlated, if at all, with Pearson coefficients of -0.09 (-0.15 to -0.02 at 95 % confidence) for stressed and 0.35 (0.05 to 0.57 at 95 % confidence) for unstressed cells. The change in cluster size due to possible diffusion in and out of the imaged volume is therefore negligible. Although some small part of the apparent change in size might thus have been caused by diffusion, this would not appreciably affect our results.

There are more clusters with smaller diffusion coefficients in the stressed cells, consistent with the higher probability for larger clusters; however, the diffusion coefficient is not strictly related to cluster size [Fig. S8b].

Diffusion coefficients computed here depend in part on the quality of tracking. To probe this, we manually checked more than 1000 clusters and their tracks and noted which clusters would be assigned differently from the algorithm. Three cases were distinguished: : (1) A track was interrupted (< 3 %); (2) A track of one cluster continued as another cluster (< 6 %); (3) Two clusters were counted as one or one cluster was counted as two (< 10 %). These errors are relatively small and we can therefore have confidence in the qualitative conclusions concerning diffusion coefficients.

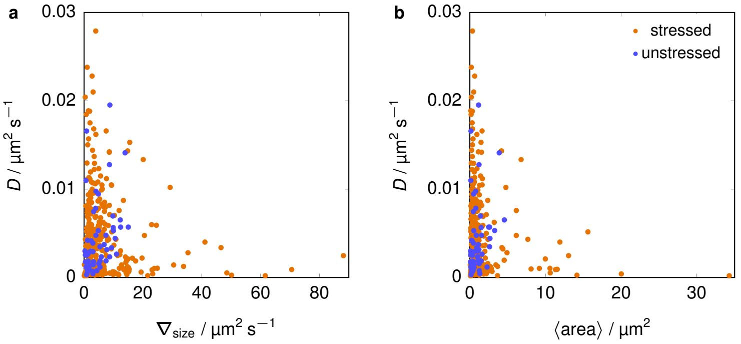

Figure S8 Effective diffusion coefficients for every cluster in living cells versus the **a** size gradients and **b** the average sizes of clusters.

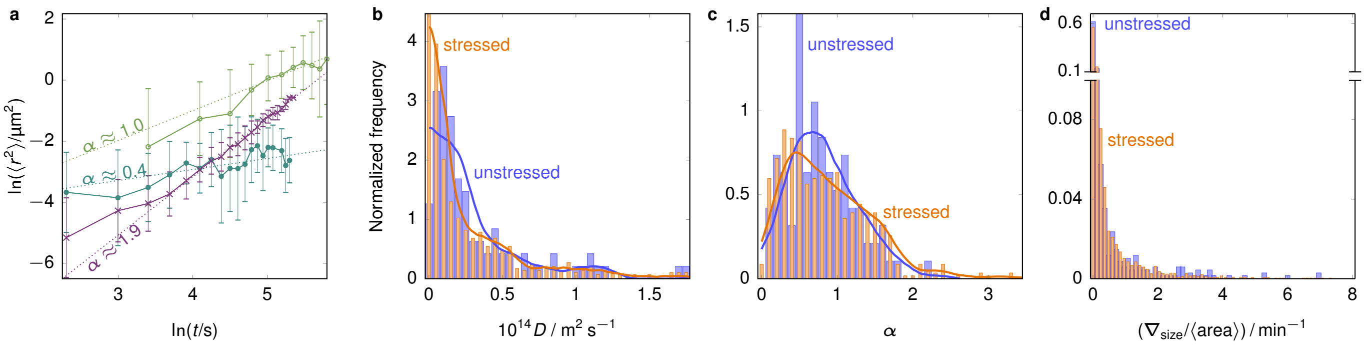

Figure S9 **a** Logarithm of the mean squared displacement of three example tracked clusters against the logarithm of the time from when the cluster was first identified. Fits to all the underlying data points give the exponent α in the generalized Einstein relation; this scaling is shown with dotted lines. Error bars show the standard deviation at each time point for the cluster in question. The numbers of data points available at each time point vary as is usual in diffusion measurements, since many more pairs are available for short time gaps between measurements than for longer ones. The number of data points for the first time point is 34 for $\alpha \approx 0.4$, 26 for $\alpha \approx 1.0$ and 30 for $\alpha \approx 1.9$, and progressively drop to 2 for the final data point reported in each case. **b** Normalized frequency of the effective diffusion coefficient for stressed and unstressed cells. **c** Normalized frequency of the diffusion exponent α for clusters in stressed and unstressed cells. In **b** and **c**, we show both histograms and a scaled kernel density estimation (Epanechnikov kernel, with reflection in the zero border for the diffusion coefficient) to reduce binning artefacts. **d** Normalized frequency of the size gradient scaled by the mean area of the cluster.

Note 9: Coalescence of clusters

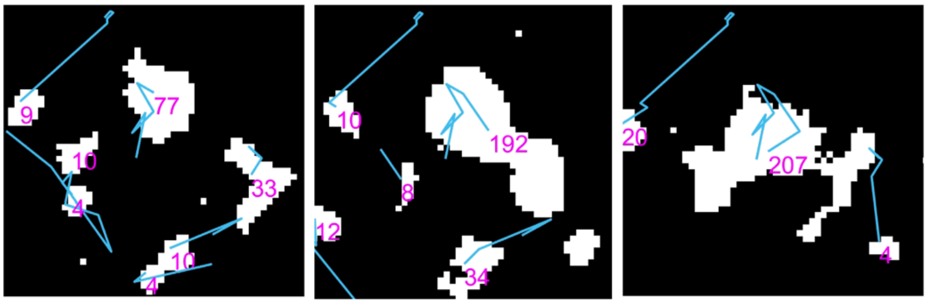

Figure S10 Possible coalescence of clusters. Taken from Supplementary Movie 5[1] at times of 120 s, 130 s and 140 s.

REFERENCES

[1]C. Lan, J. Kim, S. Ulferts, F. Aprile-Garcia, S. Weyrauch, A. Anandamurugan, R. Grosse, R. Sawarkar, A. Reinhardt, and T. Hugel, "Supplementary movies for 'Quantitative real-time in-cell imaging reveals heterogeneous clusters of proteins prior to condensation'," Zenodo (2023), 10.5281/zenodo.6946007.

[2]A. Klosin, F. Oltsch, T. Harmon, A. Honigmann, F. Jülicher, A. A. Hyman, and C. Zechner, "Phase separation provides a mechanism to reduce noise in cells," Science **367**, 464–468 (2020).

[3]P. Rawat, M. Boehning, B. Hummel, F. Aprile-Garcia, A. S. Pandit, N. Eisenhardt, A. Khavaran, E. Niskanen, S. M. Vos, J. J. Palvimo, A. Pichler, P. Cramer, and R. Sawarkar, "Stress-induced nuclear condensation of NELF drives transcriptional downregulation," Mol. Cell **81**, 1013–1026.e11 (2021).

[4]I. Arganda-Carreras, V. Kaynig, C. Rueden, K. W. Eliceiri, J. Schindelin, A. Cardona, and H. S. Seung, "Trainable Weka segmentation: a machine learning tool for microscopy pixel classification," Bioinformatics **33**, 2424–2426 (2017).

[5]G. Paul, J. Cardinale, and I. F. Sbalzarini, "Coupling image restoration and segmentation: A generalized linear model/Bregman perspective," Int. J. Comput. Vis. **104**, 69–93 (2013).

[6]A. Rizk, G. Paul, P. Incardona, M. Bugarski, M. Mansouri, A. Niemann, U. Ziegler, P. Berger, and I. F. Sbalzarini, "Segmentation and quantification of subcellular structures in fluorescence microscopy images using Squassh," Nat. Protoc. **9**, 586–596 (2014).

[7]L. Loeff, J. W. J. Kerssemakers, C. Joo, and C. Dekker, "AutoStepfinder: A fast and automated step detection method for single-molecule analysis," Patterns **2**, 100256 (2021).

[8]A. Einstein, "Über die von der molekularkinetischen Theorie der Wärme geforderte Bewegung von in ruhenden Flüssigkeiten suspendierten Teilchen," Ann. Phys.–Leipzig **322**, 549–560 (1905).
