# Supplementary material for: Quantitative real-time in-cell imaging reveals heterogeneous clusters of proteins prior to condensation
Source: Nat Commun. 2023 Aug 15;14:4831. doi: 10.1038/s41467-023-40540-2 (PMC10427612; doi:10.1038/s41467-023-40540-2)
Supplement: Supplementary file 3 — Description of Additional Supplementary Files [file 41467_2023_40540_MOESM3_ESM.pdf]

Description of Additional Supplementary Files Document

Supplementary Movie 1: Z-scan view of Fig. 1. The nuclear lamina is stained by AF647 and appears in red. Scale bars 10 µm.

Supplementary Movie 2: Three unstressed cells with NELFA-GFP. Scale bars 10 µm.

Supplementary Movie 3: Machine-learningbased tracking of NELFA-GFP regions (unstressed cells).

Supplementary Movie 4: Nine cells (stressed, with arsenic exposure) showing NELFA-GFP phase separation at low expression levels. Scale bars 10 µm. Orange rectangles mark regions of interest for the tracking of NELFAGFP regions.

Supplementary Movie 5: Machine-learningbased tracking of NELFA-GFP regions (stressed, with arsenic exposure).

Supplementary Movie 6: Time-resolved cluster size probability distribution in stressed and unstressed cells.

Supplementary Movie 7: Cluster formation in the presence of p38 inhibitor. Scale bars 10 µm.

Supplementary Movie 8: Dissolution of condensates when stress is released (the medium is exchanged) and p38 inhibitor is added. Scale bars 10 µm.
