# Supplementary material for: Quantitative real-time in-cell imaging reveals heterogeneous clusters of proteins prior to condensation
Source: Nat Commun. 2023 Aug 15;14:4831. doi: 10.1038/s41467-023-40540-2 (PMC10427612; doi:10.1038/s41467-023-40540-2)
Supplement: Supplementary file 12 — Reporting Summary [file 41467_2023_40540_MOESM12_ESM.pdf]

nature portfolio

Corresponding author(s): Aleks Reinhardt
Thorsten Hugel

Last updated by author(s): Jul 12, 2023

Reporting Summary

Nature Portfolio wishes to improve the reproducibility of the work that we publish. This form provides structure for consistency and transparency in reporting. For further information on Nature Portfolio policies, see our Editorial Policies and the Editorial Policy Checklist.

Statistics

For all statistical analyses, confirm that the following items are present in the figure legend, table legend, main text, or Methods section.

n/a	Confirmed	
☐	☒	The exact sample size (*n*) for each experimental group/condition, given as a discrete number and unit of measurement
☒	☐	A statement on whether measurements were taken from distinct samples or whether the same sample was measured repeatedly
☒	☐	The statistical test(s) used AND whether they are one- or two-sided<br>*Only common tests should be described solely by name; describe more complex techniques in the Methods section.*
☒	☐	A description of all covariates tested
☒	☐	A description of any assumptions or corrections, such as tests of normality and adjustment for multiple comparisons
☐	☒	A full description of the statistical parameters including central tendency (e.g. means) or other basic estimates (e.g. regression coefficient) AND variation (e.g. standard deviation) or associated estimates of uncertainty (e.g. confidence intervals)
☒	☐	For null hypothesis testing, the test statistic (e.g. *F*, *t*, *r*) with confidence intervals, effect sizes, degrees of freedom and *P* value noted<br>*Give P values as exact values whenever suitable.*
☒	☐	For Bayesian analysis, information on the choice of priors and Markov chain Monte Carlo settings
☒	☐	For hierarchical and complex designs, identification of the appropriate level for tests and full reporting of outcomes
☐	☒	Estimates of effect sizes (e.g. Cohen's *d*, Pearson's *r*), indicating how they were calculated

Our web collection on statistics for biologists contains articles on many of the points above.

Software and code

Policy information about availability of computer code

Data collection	Andor Solis 4.30.30034.0, ZEISS ZEN 2.6 (blue edition), ImageLab 5.2.1
Data analysis	MATLAB, FIJI. Some data analysis was performed with standard Unix command-line tools, including the following: GNU Awk 5.0.1, Gnuplot 5.2 patchlevel 8, perl 5.30.0, Python 3.8.10 (with SciPy 1.5.2, matplotlib 3.3.2, numpy 1.17.4, KDEpy 1.1.0), GNU bash 5.0.17(1), GNU sed 4.7, GNU grep 3.4, GNU findutils 4.7.0 and GNU coreutils 8.30.

For manuscripts utilizing custom algorithms or software that are central to the research but not yet described in published literature, software must be made available to editors and reviewers. We strongly encourage code deposition in a community repository (e.g. GitHub). See the Nature Portfolio guidelines for submitting code & software for further information.

Data

Policy information about availability of data

All manuscripts must include a data availability statement. This statement should provide the following information, where applicable:

- Accession codes, unique identifiers, or web links for publicly available datasets
- A description of any restrictions on data availability
- For clinical datasets or third party data, please ensure that the statement adheres to our policy

All data generated or analysed during this study are included in this published article and its supplementary files.
Supporting movies and raw data are included on Zenodo at doi:10.5281/zenodo.6946007.56

Human research participants

Policy information about studies involving human research participants and Sex and Gender in Research.

Reporting on sex and gender	N/A
Population characteristics	N/A
Recruitment	N/A
Ethics oversight	N/A

Note that full information on the approval of the study protocol must also be provided in the manuscript.

Field-specific reporting

Please select the one below that is the best fit for your research. If you are not sure, read the appropriate sections before making your selection.

☒ Life sciences ☐ Behavioural & social sciences ☐ Ecological, evolutionary & environmental sciences

For a reference copy of the document with all sections, see nature.com/documents/nr-reporting-summary-flat.pdf

Life sciences study design

All studies must disclose on these points even when the disclosure is negative.

Sample size	We have in total analysed 25 living cells (9 stressed, 3 unstressed, 13 in the p38 kinase inhibitor analysis). This ensured that the behaviour observed was similar across different cells. We report some data averaged across all cells (e.g. in the free-energy analysis), but each cell's behaviour is also reported individually to demonstrate that there is variability underpinned by a common response. In addition, we analysed 35 fixed cells for bleaching step analysis. Selection criteria are clearly specified. When we use the data of several cells, confidence intervals are given. The number of cells was chosen such that these confidence intervals are small.
Data exclusions	Live-cell movies were only taken from cells that had expression levels of NELFA-GFP such that regions of interest with well-separated clusters for tracking could be found. Only live-cells movies with arsenic exposure showing NELFA-condensation were used. Some fixed cells with non-nuclear GFP regions were excluded, because we believe that this was an artifact, since NELF condensation occures in the nuclei of HeLa cells (Rawat 2021).
Replication	All living cells experiments were performed independently. All attempts for replication were successful.<br>Only one effect was not seen in all cells, this is clearly stated in the manuscript as: "The cell-to-cell variation for this effect is high: 7 of the 13 investigated cells show full or partial disappearance of large clusters, but some also stay intact."
Randomization	Randomization was not relevant because no groups were allocated in our study.
Blinding	Blinding was not relevant because no groups were allocated in our study.

Reporting for specific materials, systems and methods

We require information from authors about some types of materials, experimental systems and methods used in many studies. Here, indicate whether each material, system or method listed is relevant to your study. If you are not sure if a list item applies to your research, read the appropriate section before selecting a response.

Materials & experimental systems

n/a	Involved in the study	
☐	☒	Antibodies
☐	☒	Eukaryotic cell lines
☒	☐	Palaeontology and archaeology
☒	☐	Animals and other organisms
☒	☐	Clinical data
☒	☐	Dual use research of concern

Methods

n/a	Involved in the study	
☒	☐	ChIP-seq
☒	☐	Flow cytometry
☒	☐	MRI-based neuroimaging

Antibodies

Antibodies used	Antibody for NELFA (1 : 500; Santa Cruz sc-23599)
Validation	The manufacturers datasheet is: https://datasheets.scbt.com/sc-23599.pdf

Eukaryotic cell lines

Policy information about cell lines and Sex and Gender in Research

Cell line source(s)	We used HeLa Flp-In T-Rex NELFA-cGFP cells. All information on the source of the cell line used is given in P.Rawat et al., Mol. Cell, 81, 1013–1026.e11 (2021)
Authentication	Transcriptome-profiling by RNA-seq.
Mycoplasma contamination	The cell lines were not tested for mycoplasma contamination.
Commonly misidentified lines (See ICLAC register)	No commonly misidentified cell line was used.
